# Multivariate data analysis identifies natural clusters of Tuberous Sclerosis Complex Associated Neuropsychiatric Disorders (TAND)

**DOI:** 10.1186/s13023-021-02076-w

**Published:** 2021-10-24

**Authors:** Petrus J. de Vries, Loren Leclezio, Sugnet Gardner-Lubbe, Darcy Krueger, Mustafa Sahin, Steven Sparagana, Liesbeth De Waele, Anna Jansen

**Affiliations:** 1 Division of Child and Adolescent Psychiatry, University of Cape Town, 46 Sawkins Road, Rondebosch, Cape Town, 7700 South Africa; 2 Department of Statistics and Actuarial Science, Stellenbosch University, Stellenbosch, South Africa; 3 Cincinnati Children’s Hospital Medical Center, University of Cincinnati College of Medicine, Cincinnati, USA; 4 Department of Neurology, Rosamund Stone Zander Translational Neuroscience Center, Boston Children’s Hospital, Boston, USA; 5 Department of Neurology, Scottish Rite for Children, The University of Texas Southwestern Medical Center, Dallas, USA; 6 Department of Pediatric Neurology, University Hospitals Leuven, Leuven, Belgium; 7 Pediatric Neurology Unit, Department of Pediatrics, UZ Brussel, Vrije Universiteit Brussel, Brussels, Belgium

**Keywords:** Tuberous Sclerosis Complex, TAND, Natural TAND clusters, Neuropsychiatric, Autism spectrum disorder, Cluster analysis, Factor analysis, Precision medicine

## Abstract

**Background:**

Tuberous Sclerosis Complex (TSC), a multi-system genetic disorder, is associated with a wide range of TSC-Associated Neuropsychiatric Disorders *(*TAND). Individuals have apparently unique TAND profiles, challenging diagnosis, psycho-education, and intervention planning. We proposed that identification of natural TAND clusters could lead to personalized identification and treatment of TAND. Two small-scale studies showed cluster and factor analysis could identify clinically meaningful natural TAND clusters. Here we set out to identify definitive natural TAND clusters in a large, international dataset.

**Method:**

Cross-sectional, anonymized TAND Checklist data of 453 individuals with TSC were collected from six international sites. Data-driven methods were used to identify natural TAND clusters. Mean squared contingency coefficients were calculated to produce a correlation matrix, and various cluster analyses and exploratory factor analysis were examined. Statistical robustness of clusters was evaluated with 1000-fold bootstrapping, and internal consistency calculated with Cronbach’s alpha.

**Results:**

Ward’s method rendered seven natural TAND clusters with good robustness on bootstrapping. Cluster analysis showed significant convergence with an exploratory factor analysis solution, and, with the exception of one cluster, internal consistency of the emerging clusters was good to excellent. Clusters showed good clinical face validity.

**Conclusions:**

Our findings identified a data-driven set of natural TAND clusters from within highly variable TAND Checklist data. The seven natural TAND clusters could be used to train families and professionals and to develop tailored approaches to identification and treatment of TAND. Natural TAND clusters may also have differential aetiological underpinnings and responses to molecular and other treatments.

## Background

Tuberous Sclerosis Complex (TSC) is a multi-system genetic disorder associated with a range of physical manifestations [1]. The main burden of the disorder is, however, linked to the neurological and neuropsychiatric features of TSC [2]. TSC-associated neuropsychiatric disorders (TAND) is seen in ~ 90% of individuals across the lifespan [3]. Previous studies suggested that each individual with TSC appears to present with their own unique profile or ‘TAND signature’ [4]. The complexity and perceived uniqueness of TAND profiles is, however, a major barrier to screening, diagnostic work-up, intervention planning, and psycho-education. Thus, identification of natural TAND clusters—predictable groupings of specific neuropsychiatric characteristics—may be a powerful strategy to reduce the perceived overwhelming heterogeneity of individual TAND profiles and resulting ‘treatment paralysis’, and could lead to the development of a personalized approach to management and treatment of individuals with TSC in clinical settings.

Machine-based data reduction methods have been used in humans to reduce the multi-dimensionality of behavioural characteristics to identify previously unrecognized clusters of behaviours. For example, in a study of the behavioural phenotype of humans with Cornelia de Lange Syndrome, categorical principal component analysis (PCA) was used as a data reduction tool and to describe relationships between a large number of behavioural variables [5]. This methodology allowed for new insights into the relationships between physical and behavioural characteristics and into genotype–phenotype correlations. In a feasibility study, we examined the viability of using multivariate data analysis techniques as a novel strategy in TSC to identify natural TAND clusters. Cluster analysis of 29 variables in 56 individuals with TSC rendered six natural TAND clusters with good face validity and significant convergence with a 6-factor exploratory factor analysis solution [6]. The natural TAND clusters identified included a ‘Scholastic’ cluster, an ‘Autism Spectrum Disorder-like’ cluster, a ‘Dysregulated behaviour’ cluster, a ‘Neuropsychological’ cluster, a ‘Hyperactive/Impulsive’ cluster, and a ‘Mixed/Mood’ cluster. The feasibility study, however, had a small sample from only two centres (South Africa and Australia) and, whilst informative from a methodological perspective, clearly required replication and expansion. Next, we therefore used the same methodology in a new sample of n = 85 individuals with TSC from 7 European countries [7]. The study also found six natural TAND clusters and replicated the majority of the earlier findings. Even though there is no consensus on sample size for cluster analysis research, we acknowledged the need for significantly larger sample size, and for evaluation of the statistical robustness and internal consistency of TAND clusters.

In this study, we used cluster and factor analysis methods in a large international sample, in search of definitive natural TAND clusters. In addition, we evaluated the robustness and internal consistency of identified natural TAND clusters.

## Methods

### Subjects

Participants for this study (n = 453) were recruited from five expert TSC centres: Cincinnati, USA (365 participants), Boston, USA (25 participants), Brussels, Belgium (25 participants), Dallas, USA (14 participants) and Leuven, Belgium (9 participants). An additional 16 participants were recruited through Tuberous Sclerosis International (TSCi). To be eligible, participants had to meet clinical criteria for TSC [1]. Anonymized data deliberately included participants with a wide age and ability range. All procedures contributing to this work complied with the ethical standards of the relevant national and international committees on human experimentation and with the Helsinki Declaration of 1975, as revised in 2008. The protocol was peer-reviewed in the Department of Psychiatry at the University of Cape Town and submitted for ethical approval by the Faculty of Health Sciences, Human Research Ethics Committee (Ethics Ref 340/2015). Additional study sites obtained ethical approval or waivers from their respective HREC/IRB bodies.

### Procedures

The TAND Checklist was administered to parents and caregivers of individuals with TSC by the resident TSC coordinator and/or treating physician. The TAND Checklist is a short pen-and-paper checklist that captures the high frequency neuropsychiatric difficulties in TSC. It was developed in partnership with family and professional stakeholders in the TSC community. It typically takes about 10–20 min to complete. For details about TAND and the TAND Checklist, including a downloadable version of the Checklist, please see de Vries et al. [3].

### Data analysis

**Fig. 1:**
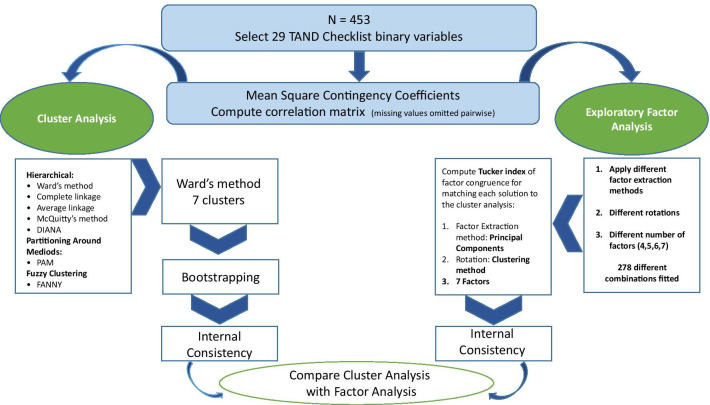
Schematic overview of data analysis in the study

Statistical analysis was performed on anonymized TAND Checklist data using a series of steps as outlined below and in Fig. 1.

*STEP 1. Select TAND checklist variables*

The following sections of the TAND Checklist were included in the analysis: Section 3, behavioural challenges (19 questions/variables); Section 5, academic skills (4 variables); and Section 7, neuropsychological skills (6 variables). This equated to 29 dichotomous TAND variables.

*STEP 2. Compute correlation matrix*

Mean squared contingency coefficient [8] was used to compute a correlation matrix for the 29 binary variables selected from the TAND Checklist. Where missing values were present, these were omitted pairwise in correlation computations.

*STEP 3. Cluster analysis*

Several clustering solutions were compared. Hierarchical clustering methods provide a clustering tree visually representing the merging of TAND variables and suggesting a suitable number of clusters. Hierarchical clustering methods including complete linkage, average linkage, Ward’s method and McQuitty’s method were applied with the R functions hclust() in base R [9]. Although hierarchical clustering has often been used with great success, the algorithm is fairly naïve and some more recent methods in the R package cluster [10] were therefore also investigated. The FANNY (fuzzy clustering) method applied to the data allocates a probability for belonging to each cluster rather than simply allocating each item to a single cluster. DIANA is a divisive analysis hierarchical clustering method, whereas the other hierarchical methods are all agglomerative.

*STEP 4. Bootstrapping*

Following cluster analysis, 1000-fold bootstrapping [11] was applied to assess the statistical robustness of the clustering solution. Based on the bootstrap sample of patients, a squared contingency coefficient correlation matrix was computed, and hierarchical cluster analysis with Ward’s method was performed. The number of clusters was fixed at 7 and a bootstrap replicate clustering solution was obtained. In order to assess the robustness or stability of the original observed clustering solution, the number of times each pair of variables clustered together was calculated.

*STEP 5. Exploratory factor analysis*

After suitable clustering solutions were obtained and bootstrapping applied, exploratory factor analysis was employed with the fa() R function in the package psych [12]. The factor analysis was also performed on the mean squared contingency coefficient correlation matrix. All the different options of factor extraction and rotation available in the fa() function were investigated. These combinations were applied to solutions with between four and seven factors. In order to find the factor solution that best matched the cluster analysis solution, the Tucker index of factor congruence [13] was used. Optimal rotations were achieved with Orthogonal Procrustes Analysis [14] and the overall congruence summarised by the sum of the diagonal values. For cases other than 6 factors, the congruence matrix was padded with zeros to obtain a square matrix before rotation. Full algebraic details are available in the supplemental data of Leclezio, Gardner-Lubbe & de Vries [6].

*STEP 6. Test internal consistency*

Reliability analysis [15] was used to test the internal consistency of the TAND variables both the clusters identified and factors generated and in the final proposal for clusters.

*STEP 7. Compare cluster analysis findings with exploratory factor analysis results*

Here we compared the data-driven cluster solutions with the exploratory factor analysis in order to examine similarities and differences of the two approaches.

*Step 8. Description of final natural clusters*

Finally, we integrated all results to generate a final set of natural TAND clusters.

## Results

### Cluster analysis

**Fig. 2:**
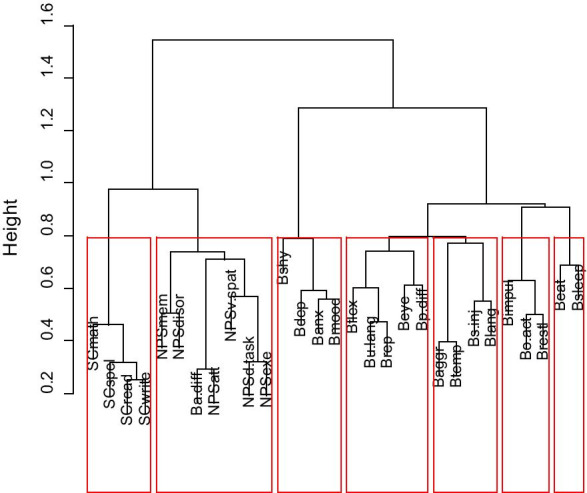
Cluster analysis findings of the study (n = 453). The figure shows the dendrogram illustrating the 7 natural TAND clusters generated with Ward’s cluster analysis method

Hierarchical clustering with Ward’s method produced a seven-cluster solution. A dendrogram of the Ward cluster analysis shows detail of the natural clustering of the TAND variables examined (Fig. 2).

The first cluster included difficulties with mathematics, spelling, writing, and reading suggesting a natural ‘Scholastic’ cluster. The second cluster included memory difficulties, getting disorientated, attention difficulties at both a behavioural and neuropsychological level, difficulty with visuo-spatial tasks, dual-task difficulties, and executive skills difficulties. These suggested a natural ‘Neuropsychological’ cluster. The third cluster included extreme shyness, depressed mood, anxiety, and mood swings, suggesting a natural ‘Mood/Anxiety’ cluster. The fourth cluster included inflexibility, unusual language (repeating words or phrases over and over again), repetitive behaviours, poor eye contact, and peer difficulties, suggesting an autism spectrum disorder (ASD)-like cluster. The fifth cluster included aggressive outbursts, temper tantrums, self-injury, and absent or delayed onset of language. The first three items supported a natural ‘Dysregulated behaviour’ cluster, but the delayed language item did not overtly seem to fit the natural cluster. The sixth cluster contained impulsivity, overactivity, and restlessness, suggesting a natural ‘Overactive/Impulsive’ cluster. The seventh cluster contained two biological items—difficulties with eating and sleep-related problems. We refer to this as the ‘Eat/Sleep’ cluster.

### Bootstrapping

**Fig. 3.**
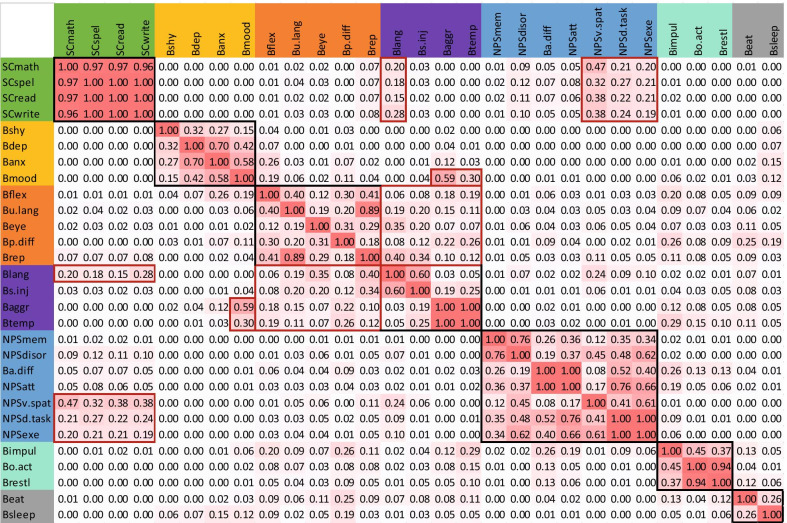
1000-Fold Bootstrapping applied to WARD’s cluster analysis. Results are expressed as the proportion of time that two individual items cluster together

Bootstrapping results are shown in Fig. 3. Black boxes are shown around the clusters as shown in the dendrogram (see Fig. 2). Red boxes are shown around other items that showed relationships between variables outside clusters. The Scholastic cluster was very stable with all four items clustering together 96% of the time. Three neuropsychological items and absent/delayed language showed association with the scholastic cluster (Visuo-spatial skills, 32–47%; Dual-tasking, 21–27%; Executive skills, 19–21%; Absent/Delayed language, 15–28%). In the Neuropsychological cluster, items showed bootstrap values ranging from 100% (behavioural and neuropsychological attention difficulties; dual-tasking and executive skills), to 76% (Memory and Disorientation), while other pairings were less stable (Behavioural and neuropsychological attention deficits in relation to visuo-spatial deficits, 8–17%). Items in the Overactive/Impulsive cluster clustered together 94% (overactive, restless), 45% (impulsivity, overactive) and 37% of the time (impulsivity, restlessness). In the Mood/Anxiety cluster extreme shyness showed bootstrapping values between 15 and 32%, depressed mood 32–70%, anxiety 27–70%, and mood swings 15–58%. Depressed mood also associated with aggression and temper tantrums 59% and 30% of the time, respectively. The fifth cluster, Dysregulated behaviour, showed that temper tantrums and aggressive outbursts clustered together 100% of the time and that self-injury and absent/delayed language clustered together 60% of the time. Self-injury clustered with aggression and temper tantrums 19–25% of the time, but absent/delayed language showed very low bootstrapping values (3–5%). Instead, absent/delayed language showed higher bootstrapping values with items in other clusters, particularly the scholastic (15–28%) and ASD-like cluster (6–40%). The ASD-like cluster items ranged between 12 and 89% of which unusual language and repetitive behavior showed the highest bootstrapping values (89%). The two items of the Eat/Sleep cluster clustered together 26% of the time.

### Factor analysis

**Fig. 4:**
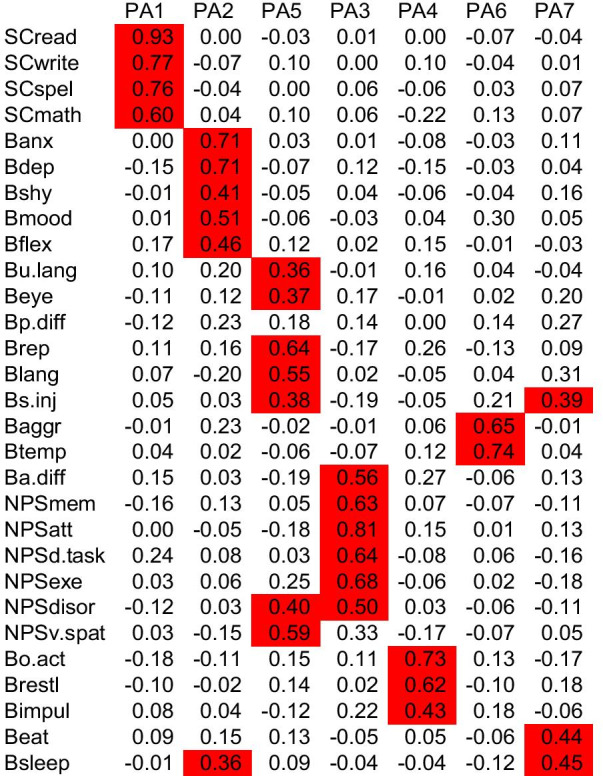
Factor analysis findings of the study (n = 453). The figure shows the seven-factor solution generated by exploratory factor analysis (EFA). PA = principal axis

Factor loadings (cut-off > 0.35) from exploratory factor analysis are shown in Fig. 4. The factor analysis solution that most closely matched Ward’s hierarchical cluster analysis was the principal components factor extraction method with clustering method rotation. Results supported a seven-factor solution very similar to the cluster solution outlined above. Three items cross-loaded onto more than one factor. These were self-injurious behavior (ASD-like factor and Eat/Sleep factor), sleeping difficulties (Eat/Sleep factor and Mood/Anxiety factor), and disorientation (ASD-like factor and the Neuropsychological factor).

### Internal consistency

Five clusters had Cronbach alpha values ≥ 0.7 indicating good to excellent internal consistency: Scholastic (0.97), Neuropsychological (0.87), ASD-like (0.76), Dysregulated behaviour (0.74), and Overactive/Impulsive (0.70). The remaining 2 clusters had lower alpha values: Mood/Anxiety (0.69) and Eat/Sleep (0.48). Similarly, in the factor analysis solutions 6 factors showed good to excellent internal consistency: Scholastic (0.97), Neuropsychological (0.86), ASD-like (0.79), Dysregulated behaviour (0.75), Mood/Anxiety (0.74) and Overactive/Impulsive (0.70). Only the Eat/Sleep factor scored < 0.7 with an alpha = 0.54, suggesting poor internal consistency.

### Comparison of cluster analysis and factor analysis findings

**Fig. 5:**
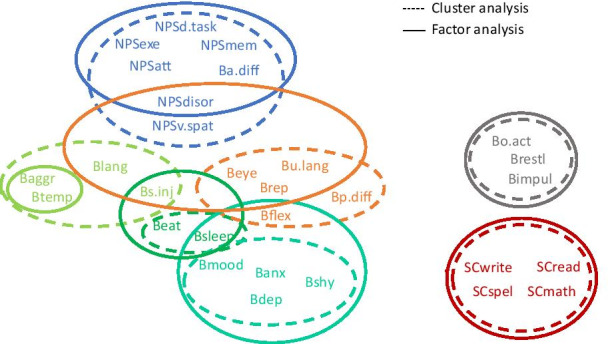
Relationship between natural TAND Clusters and Factor Analysis. Results show two highly distinct clusters (Scholastic and Overactive/Impulsive), three fairly distinct clusters (Mood/Anxiety, Neuropsychological, Eat/Sleep), and two clusters showing more significant cross-loading (ASD-like and Dysregulated behaviour)

Factor analysis confirmed a profile similar to cluster analysis, but with some slight variance between clusters and factors (Fig. 5). Cluster and factor analysis showed the ‘Overactive/Impulsive’ and ‘Scholastic’ clusters to be clearly distinct, the ‘Mood/Anxiety’ and ‘Neuropsychological’ clusters to be fairly distinct, while the other three clusters/factors showed more evidence of cross-loading between items in different clusters/factors.

### Integrated findings of natural TAND clusters

In this study three different sets of ‘data-driven outputs’ were generated – cluster analysis, factor analysis, and bootstrapping of the cluster analysis. They all represented related but different components of the multivariate data analysis performed here. All except two items presented convergent findings in terms of clusters, bootstrapping and factors. The two outlier items (delayed language and self-injury) therefore required a systematic review of the three data outputs and a final decision required expert review.

Absent/delayed language was linked with self-injury in the dysregulated behaviour cluster during hierarchical cluster analysis. However, bootstrapping values for the item were low inside the cluster (3–5%), but higher with items in the scholastic and ASD-like cluster. In factor loading, absent/delayed language showed the highest loading with the ASD cluster items. After review of all data, the item was therefore moved to the ASD-like cluster. Self-injury was retained in the dysregulated behaviour cluster.

Taking together all results, we propose seven natural TAND clusters. Table 1 shows the integrated natural clusters, component items and internal consistency for each natural TAND cluster (see Table 1).

**Table 1:** Seven natural TAND clusters identified in this study

Natural TAND cluster	No. of items	TAND checklist items	Internal consistency (alpha)
Scholastic	4	Reading Writing Spelling Mathematics	0.97^a^
Neuropsychological	7	Memory Disorientation Attention difficulties (behaviour) Neuropsychological attention deficits Visuo-spatial Dual-tasking Executive skills	0.87^a^
Autism Spectrum Disorder-Like	6	Inflexible Unusual language Repetitive behaviour Poor eye contact Peer difficulties Delayed language	0.79^a^
Dysregulated behaviour	3	Aggressive outbursts Temper tantrums Self-injury	0.73^a^
Overactive/Impulsive	3	Overactive Impulsive Restless	0.70^a^
Mood/Anxiety	4	Mood swings Anxiety Depressed mood Extreme shyness	0.69
Eat/Sleep	2	Eating difficulties Sleep difficulties	0.48

The table shows the clusters with items contained and internal consistency of each cluster

^a^Cronbach alpha ≥ 0.7 indicates good internal consistency

## Discussion

TSC-Associated Neuropsychiatric Disorders (TAND) have largely gone undiagnosed and untreated despite affecting 90% of individuals with TSC. This has been attributable to lack of awareness, and lack of expertise in TAND, but most fundamentally, the overwhelming uniqueness of TAND profiles. We proposed that the identification of naturally occurring TAND clusters may improve identification and intervention [4]. In a feasibility study, we showed that a data-driven approach was able to identify natural clusters of TAND [6], and these findings were replicated in a second study [7]. However, findings required larger-scale replication and extension, particularly to evaluate the robustness of proposed natural TAND clusters. In this study, various cluster analysis techniques and exploratory factor analysis was applied in a large and diverse international sample (n = 453). In addition, bootstrapping and internal consistency analyses were performed.

Results identified seven natural TAND clusters, and bootstrapping showed clusters to be reasonably stable. The scholastic cluster showed the highest robustness in terms of replicability on bootstrapping, while other clusters showed a degree of agreement, suggesting that, with the exception of one item, the identified cluster solution as shown in the dendrogram (Fig. 2) is sufficiently replicable and stable to use in next-step work. The one problematic item in terms of cluster placement (absent/delayed language) was moved from the dysregulated cluster to the ASD-like cluster after expert statistical and clinical review of constructs. Exploratory factor analysis showed that a 7-factor solution mapped well onto the majority of clusters. The Scholastic, Neuropsychological, Overactive/Impulsive, and Eat/Sleep clusters showed good agreement between clusters and factors, but significant cross-loading was observed between the ASD-like, Dysregulated behaviour and Mood/Anxiety clusters. Internal consistency for clusters and factors was good to excellent for 5 of the original 7 clusters generated (except for the Mood/Anxiety and Eat/Sleep clusters) and for 6 of the 7 factors identified (except for the Eat/Sleep factor). In slight contrast to the feasibility studies that suggested 6 natural TAND clusters [6, 7], this larger-scale study identified 7 TAND clusters. In this study, the two biological (vegetative) items (sleeping/eating) grouped together, whereas in the feasibility studies they were incorporated into the ASD-like cluster (difficulties with eating) and the Mood/Anxiety cluster (sleep difficulties). The remaining 6 clusters, however, were remarkably similar to the findings from the small-scale feasibility and replication studies [6, 7].

## Seven natural TAND clusters

As outlined in Table 1, integration of the multivariate findings led to our proposal of 7 natural clusters for further validation and potential implementation.

Cluster 1. Scholastic cluster

The first of the seven natural TAND clusters identified is a ‘Scholastic’ cluster indicating difficulties relating to reading, writing, spelling and mathematics. The items in the Scholastic cluster (rendered by both cluster analysis and factor analysis) showed high bootstrapping, very high factor loadings and alpha scores, indicating the close relationship and reliability between items. Findings highlight the need for assessment in this cluster if an individual shows signs of difficulty across any one of the four items. Academic difficulties are a common concern in TSC [2, 3, 16, 17] and not only affect school-aged children, but also have long-term consequences in adulthood.

Cluster 2. Overactive/Impulsive cluster

Both cluster analysis and factor analysis includes overactivity, restlessness, and impulsivity in this cluster. Bootstrapping and internal consistency were high, indicative of the reliability of items and how they group together. This cluster appears clinically meaningful given the high rates of Attention Deficit Hyperactivity Disorder (ADHD) reported in TSC [16–18]. However, it is of interest that the cluster did not include attentional difficulties, which were grouped in the neuropsychological cluster. This may suggest ADHD in TSC to be more typically of the ‘predominantly hyperactive/impulsive subtype’ or could suggest that there may be differential pathways to the attentional and hyperactive/impulsive deficits seen in TSC.

Cluster 3. Neuropsychological cluster

This cluster includes memory deficits, disorientation, neuropsychological attention deficits as well as attention deficits in daily life, dual task deficits, executive deficits, and visuo-spatial deficits. Whilst visuo-spatial deficits were grouped within the ASD factor, cluster analysis grouped visuo-spatial deficits with the other neuropsychological skills. Bootstrapping supported the clustering with neuropsychological skills but confirmed a frequent co-occurrence with the scholastic cluster. Based on the existing TSC literature, the cluster maps very well onto the high rates of a range of neuropsychological attentional, executive, and memory deficits reported [16, 17, 19–21].

Cluster 4. Mood/anxiety cluster

Four items are included in this cluster – anxiety, depressed mood, mood swings and extreme shyness. We observed that factor analysis included inflexibility and sleep-related problems with the four other items. However, bootstrapping classified these two items in the mood/anxiety cluster only 12–15% (sleep) and 19–26% (inflexibility) of the time. Given the cluster analysis and bootstrapping observed, inflexibility was therefore retained with ASD-like features, and ‘sleep difficulties’ with the Eat/Sleep cluster. The four mood/anxiety items (mood swings, anxiety, depressed mood, extreme shyness) are commonly seen in children and adults with TSC [16, 17, 17, 18, 22].

Cluster 5. Dysregulated behaviour cluster

The dysregulated behaviour cluster includes aggressive outbursts, temper tantrums and self-injurious behaviour. Cluster analysis also included absent/delayed language in the cluster, but, as outlined earlier, bootstrapping did not support the robustness of this item in the cluster. One of the biggest concerns to families is the high rate of ‘behaviors that challenge’ seen in TSC specifically with regards to aggression and temper tantrums, self-injury and damage to property [16–18, 23, 24]. It was therefore of interest that a specific and distinct cluster of dysregulated behaviours was identified here.

Cluster 6. Autism Spectrum Disorder (ASD)-like cluster

This natural TAND cluster includes six items—inflexibility, unusual language, repetitive behaviour, poor eye contact, peer difficulty and delayed/absent language. As outlined above, initial cluster analysis did not include absent/delayed language in the ASD-like cluster, but bootstrapping and factor analysis suggested these characteristics to be more likely to co-occur with ASD-like rather than with other TAND behaviours. TSC is one of the medical conditions most strongly associated with ASD and the symptoms of ASD in TSC seems to map very well with symptoms observed in non-syndromic ASD [2, 3, 16–18, 25, 26]. It was therefore of interest to see the natural emergence of an ASD-like cluster of behaviours from a clinical perspective.

Cluster 7. Eat/sleep cluster

This cluster includes eating and sleeping difficulties. Given that sleeping and eating are fundamental biological/vegetative functions it was not surprising to see them cluster together. High rates of sleep problems have been reported in individuals with TSC [27], and deficits in circadian rhythm are now described in animal models of this disorder [28]. This cluster was not identified in the feasibility study, but this much larger sample suggested that these concerns group together. Importantly, both sleep and eating difficulties cross-load with other clusters, underlining the fact that they often co-occur with other neuropsychiatric difficulties. However, the fact that they cluster independently suggests the need to investigate these in their own right, not only in the context of other so-called co-morbid conditions.

## Study limitations

Firstly, anonymized TAND Checklist data were used here to identify potential natural TAND clusters, and no other sources of information that may be relevant in cluster analysis or factor analysis, such as clinical evaluations or neuropsychological assessments, were included. We acknowledge that it is therefore theoretically possible that other clusters may be identified using different kinds of multi-level data. However, we specifically wanted to use the TAND Checklist for this purpose, given that it is a simple, yet systematic and freely available tool that could easily be implemented in real-life settings around the globe. Pilot validation of the TAND Checklist [29] indicated that the TAND Checklist was a valid tool in extrapolating multi-level neuropsychiatric manifestations in TSC. It would be important to include external validation of the natural TAND cluster findings based on TAND Checklist data in relation to expert clinical data as a logical next step. Secondly, we acknowledge that all the data were ‘lifetime’ data. We have therefore to date not examined the developmental pattern of natural TAND clusters, which may have a more dynamic nature than captured here. However, our findings should allow longitudinal examination of natural TAND clusters in future large-scale studies. This should also include examination of the association between age, gender, intellectual ability and other potential correlates of TAND. Thirdly, the current TAND Checklist collects data in a dichotomous fashion. The study was therefore not able to explore the subtleties of severity that may be important to examine in natural TAND clusters in future. Development of a quantified version of the TAND Checklist is currently underway.

## Conclusions

Based on the largest collection of TAND Checklist data to date, our analyses generated seven natural TAND clusters that were statistically robust and had good clinical face validity. We therefore propose the identification of these clusters to be a useful first step as a guide to further assessment and treatment options in clinical practice. Next steps could include the development of targeted teaching and training of professionals and individuals with TSC about the seven natural TAND clusters, and identification of appropriate evidence-based resources and interventions that map onto these clusters. At a scientific level, we propose that identification of these naturally-occurring clustering of the neuropsychiatric phenotype of TSC may be the first step towards a more dimensional, data-driven approach to the study of the etiology and treatments (molecular and otherwise) [2, 17, 30] of individuals with TSC and related neurodevelopmental disorders.

## Data Availability

The data that support the findings of this study are available from the corresponding author [PdV] upon reasonable request. Full algebraic details of analysis methodologies are available in the supplemental data of Leclezio, Gardner-Lubbe & de Vries [6].
